# A Novel Adult Murine Model of Typical Enteroaggregative *Escherichia coli* Infection Reveals Microbiota Dysbiosis, Mucus Secretion, and AAF/II-Mediated Expression and Localization of β-Catenin and Expression of MUC1 in Ileum

**DOI:** 10.3389/fcimb.2022.885191

**Published:** 2022-05-30

**Authors:** Nadia Moran-Garcia, Catalina Lopez-Saucedo, Adriana Becerra, Mario Meza-Segura, Felipe Hernandez-Cazares, Jair Guerrero-Baez, Silvia Galindo-Gómez, Víctor Tsutsumi, Michael Schnoor, Alfonso Méndez-Tenorio, James P. Nataro, Teresa Estrada-Garcia

**Affiliations:** ^1^ Department of Molecular Biomedicine, CINVESTAV-IPN, Mexico City, Mexico; ^2^ Department of Cell Biology, CINVESTAV-IPN, Mexico City, Mexico; ^3^ Department of Infectomics and Molecular Pathogenesis, CINVESTAV-IPN, Mexico City, Mexico; ^4^ Departamento de Bioquímica, Escuela Nacional de Ciencias Biológicas, Instituto Politécnico Nacional, Mexico City, Mexico; ^5^ Department of Pediatrics, University of Virginia School of Medicine, Charlottesville, VA, United States

**Keywords:** enteroaggregative, *E. coli*, mouse model, mucus production, biofilm formation, aggregative adherence fimbria/II, MUC1, beta-catenin

## Abstract

Typical enteroaggregative *Escherichia coli* (tEAEC) is a diarrheagenic *E. coli* pathotype associated with pediatric and traveler’s diarrhea. Even without diarrhea, EAEC infections in children also lead to increased gut inflammation and growth shortfalls. EAEC strain’s defining phenotype is the aggregative adherence pattern on epithelial cells attributable to the aggregative adherence fimbriae (AAF). EAEC only causes diarrhea in humans; therefore, not much is known of the exact intestinal region of infection and damage or its interactions with intestinal enterocytes *in vivo* and *in situ*. This study aimed to develop a new tEAEC mouse model of infection, characterize the microbiota of infected mice, and evaluate *in situ* the expression of host adherence and surface molecules triggering EAEC infection and the role of the EAEC AAF-II in adherence. Six-week-old C57BL/6 mice, without previous antibiotic treatment, were orally challenged with EAEC 042 strain or EAEC 042 AAF-II mutant (ΔAAF/II) strain, or DAEC-MXR strain (diffusely adherent *E. coli* clinical isolate), and with saline solution (control group). Paraffin sections of the colon and ileum were stained with H&E and periodic acid-Schiff. ZO-1, β-catenin, MUC1, and bacteria were analyzed by immunofluorescence. EAEC-infected mice, in comparison with DAEC-MXR-infected and control mice, significantly lost weight during the first 3 days. After 7 days post-infection, mucus production was increased in the colon and ileum, ZO-1 localization remained unaltered, and morphological alterations were more pronounced in the ileum since increased expression and apical localization of β-catenin in ileal enterocytes were observed. EAEC-infected mice developed dysbiosis 21 days post-infection. At 4 days post-infection, EAEC strain 042 formed a biofilm on ileal villi and increased the expression and apical localization of β-catenin in ileal enterocytes; these effects were not seen in animals infected with the 042 ΔAAF/II strain. At 3 days post-infection, MUC1 expression on ileal enterocytes was mainly detectable among infected mice and colocalized with 042 strains on the enterocyte surface. We developed a novel mouse model of EAEC infection, which mimics human infection, not an illness, revealing that EAEC 042 exerts its pathogenic effects in the mouse ileum and causes dysbiosis. This model is a unique tool to unveil early molecular mechanisms of EAEC infection *in vivo* and *in situ*.

## Introduction

Enteroaggregative *Escherichia coli* (EAEC) is one of the six diarrheagenic *E. coli* pathotypes (Harrington et al., 2006; Estrada-Garcia and Navarro-Garcia, 2012; Modgil et al., 2020). EAEC strain’s defining phenotype is the aggregative adherence pattern on epithelial cells, attributable to the aggregative adherence fimbriae (AAF) (Estrada-Garcia and Navarro-Garcia, 2012; Modgil et al., 2020). The AggR regulon, which defines typical EAEC (tEAEC) strains, was first described in the archetype EAEC strain 042 (serotype O44:H18) isolated from a Peruvian child with diarrhea (Czeczulin et al., 1997; Harrington et al., 2006). AggR is a member of the AraC/XylS family of bacterial transcriptional activators that control the expression of a plethora of virulence factors; in strain 042, it is encoded on both the bacterial chromosome and the EAEC-specific aggregative adherence plasmid (pAA) (Estrada-Garcia and Navarro-Garcia, 2012). AggR activates the expression of factors important in colonization, such as dispersin (AAP), allowing EAEC strains to disperse through the mucus and reach the enterocyte surface, AAF variants I–V, which mediate adherence *in vitro* to human epithelial cells and probably promote intestinal colonization, and the Aai-type VI secretion system (Dudley et al., 2006), important in bacterial pathogenesis (Jønsson et al., 2017; Navarro-Garcia et al., 2019; Prieto et al., 2021). In addition, tEAEC has a variable number of SPATEs (serine protease autotransporters of Enterobacteriaceae), including Pic (a protein involved in intestinal colonization), which is involved in colonization and hypersecretion of mucus, a hallmark of EAEC pathogenesis (Navarro-Garcia et al., 2010).

tEAEC infection causes enteric disease in diverse clinical settings, including acute diarrheal illness, particularly in infants and young children from less developed regions of the world (Estrada-Garcia and Navarro-Garcia, 2012; Patzi-Vargas et al., 2015; Modgil et al., 2020), and severe diarrhea among Peruvian infants and adults infected with HIV (Garcia et al., 2010; Medina et al., 2010), and is the second most common cause of traveler’s diarrhea worldwide (Paredes-paredes et al., 2011; Jensen et al., 2014). In patients, EAEC causes watery mucoid diarrhea, which may contain fecal leukocytes and/or lactoferrin and can be especially severe among infants and adult travelers (Jiang et al., 2003; Acosta et al., 2016; Meza-segura et al., 2020). Notably, a unique characteristic of tEAEC infection is its association even in the absence of diarrhea, with growth shortfalls characterized by a significantly greater height-for-age Z-score (HA-Z) decline (*p* < 0.05) in children from less developed areas; it may also cause malnutrition (Opintan et al., 2010; Acosta et al., 2016). Furthermore, the development of irritable bowel syndrome (IBS) has been documented following traveler’s diarrhea caused by EAEC (Dupont et al., 2010), and IL-8 polymorphism has been associated with host diarrheal illness or EAEC infection (Jiang et al., 2003). Therefore, tEAEC infection has become an important public health problem among children from less developed regions of the world and travelers, revealing the necessity to develop animal models to characterize the gut region where EAEC exerts its pathogenesis, among other issues (Philipson et al., 2013). The aims of the present study were to develop a mouse model of tEAEC infection using C57BL/6 adult mice orally inoculated with the archetype EAEC 042 strain, to characterize EAEC infection *in vivo* and *in situ*, to evaluate the expression of ZO-1 tight junctions and β-catenin adherence junction and the role of AAF/II fimbriae in β-catenin and MUC1 expression during infection.

## Materials and Methods

### Mice

Wild-type C57BL/6 mice that were 6 to 7 weeks old, derived from a C57BL/6 background (Jackson Laboratory, Bar Harbor, ME, USA), were kept under controlled conditions of a temperature of 25°C and a 12-h light/dark cycle in autoclaved microisolator filtered cages, with sterile bedding. Mice were fed with irradiated control diet (Research Diets, D09051102) or an autoclavable rodent breeder diet (LabDiet, St. Louis, MO, USA; 50I3) with *ad libitum* access to food and sterilized water. Before the infection, the mice were free of *E. coli*, confirmed by fecal samples cultured on MacConkey agar plates.

### Bacterial Strains and Inoculation

The EAEC strains used in this study were the wild-type EAEC 042 and a derivative mutant for AAF/II (ΔAAF/II), which was provided by Dr. James Nataro Laboratory, University of Virginia (Charlottesville, VA, USA). *E. coli* strains HS and 3030 (carrying *afa* gene) reference strains plus DAEC-MXR strain, isolated from a 1-year-old Mexican child with diarrhea, were included as controls.

### Infection and Experimental Design

Before inoculation, the intestinal pH of C57BL/6 mice was neutralized with NaHCO_3_ (0.35 M); then after 15 min, mice were orally inoculated *via* gavage needle with 9.5 × 10^9^, 1 × 10^10^, or 5 × 10^9^ colony-forming unit (CFU) of EAEC 042 or control strains in 100 μl of vehicle buffer (saline solution 0.8%), as previously described by Bernal-Reynaga et al. (2013). In addition, another set of mice was orally inoculated with the vehicle buffer.

### Determination of Colony-Forming Unit per Gram of Feces

Every day, body weight was measured and fecal samples were collected until the day mice were sacrificed by cervical dislocation under chloroform inhalation. Briefly, collected fecal samples were weighed and resuspended in phosphate-buffered saline (PBS); then logarithmic 10^−1^–10^−3^ serial dilutions were prepared, plated onto MacConkey agar plates, and incubated at 37°C for 24 h Bernal-Reynaga et al. (2013). The next day, colonies were counted, and their concentration was determined and expressed in CFU/g of feces. In order to verify that the recovered bacteria were EAEC 042, a multiplex PCR targeting *aggR*, *aatA*, and *aap* genes was performed on the isolated colonies (Cerna et al., 2003).

### Quantification of *Escherichia coli* on Intestinal Tissues

At 7 days post-infection, ileum and colonic mucosal tissues were collected, weighed, rinsed with PBS, and then frozen. Tissue DNA was extracted with QuickGene SP kit DNA (FUJIFILM, Tokyo, Japan). The tissue bacterial load was determined by quantitative real-time PCR, screening for *aaiC* gene (F:5′ ATTGTCCTCAGGCATTTCAC 3′ and R:5′ ACGACACCCCTGATAAACAA 3′) (Panchalingam et al., 2012) in a StepOne Thermo Fisher (Waltham, MA, USA) thermocycle with the following conditions: 50°C 2 min, 95°C 5 min (1 cycle); 95°C 45 s, 58°C 20 s, 72°C 20 s (38 cycles). The melting curve was set at 95°C 15 s, 68°C 1 min, and 85°C 15 s.

### Histology

Excised ileum and colonic mucosal tissue specimens were fixed with 100% ethanol or Carnoy’s solution (60% ethanol, 30% chloroform, and 10% acetic acid), embedded in paraffin, and cut into 5-µm sections. Sections were stained with H&E and periodic acid-Schiff (PAS) and by immunofluorescence and immunochemistry staining. Mucosa and villus histological evaluations were performed in H&E-stained tissues, and sections stained with PAS were used to evaluate mucus production. Images were processed using ImageJ 2.0.0-rc-65/1.52i software.

### Immunofluorescence

Fixed tissues were deparaffinized/dehydrated, and the slides were then treated for antigen retrieval. Briefly, a staining dish containing citrated buffer (0.01 M, pH 6) was introduced into a preheated water bath until the water reached a temperature of 95°C–100°C, then the slides were immersed in the staining dish, and the lid was placed loosely, allowing the slides to incubate for 20 min. Finally, slides were removed and left at room temperature.

For immunofluorescence, slides were incubated for 2 h with PBS containing 5% bovine serum albumin (BSA) to block unspecific binding. Immunolabeling with primary antibodies was performed overnight at 4°C followed by washing and incubation with species-specific fluorescently labeled secondary antibodies conjugated with Alexa 488 (A11008; Invitrogen, Carlsbad, CA, USA) and Alexa 568 (A11077; Invitrogen, CA, USA). The following primary antibodies were used: Alexa 568-labeled phalloidin, anti-ZO-1 (61-7300; Invitrogen, CA, USA), anti-β-catenin ((H-102): sc-7199; Santa Cruz Biotechnology, Texas, USA), anti-MUC1 (ab15481; Abcam, Boston, MA, USA), and anti-EAEC 042 ΔAAF/II (home produced).

Preparations were mounted in a fluorescent mounting medium with DAPI (Vectashield) and examined using a laser scanning confocal imaging system (Leica Biosystems, Wetzlar, Germany; SP8, confocal) at the National Laboratory of Experimental Services (LANSE), CINVESTAV-IPN. Images were processed using ImageJ 2.0.0-rc-65/1.52i software.

### 16S rRNA Sequencing and Microbiota Analysis

From pool stools of the same three mice collected 1 day before EAEC 042 infection and 21 days after infection, DNA was extracted using a QIAamp DNA stool kit (Qiagen, Hilden, Germany) following the manufacturer’s protocol. However, the last column elution was done using 200 μl of nuclease-free water. DNA was quantified using a NanoDrop2000 spectrophotometer (Thermo Fisher Scientific, Waltham, MA, USA), and DNA integrity was verified by PCR assay as previously described by Simon et al. (2014), using primers that amplify a 107-bp fragment of the 16S rRNA gene of *E. coli* (Simon et al., 2014).

DNA obtained from the stool samples was used for the amplification of the 16S rRNA gene with the primers targeting regions V3–V5 (357-F: 5′-CTCCTACGGGAGGCAGCAG-3′ and CD-R: 5′-CTTGTGCGGGCCCCCGTCAATTC-3′). Sequencing libraries were prepared following the 16S Metagenomic Sequencing Libraries Preparation guide and were sequenced on the Illumina-MiSeq platform at the CINVESTAV-Langebio Laboratory to generate tagged paired-end reads. The obtained sequences have been submitted to the NCBI database under accession number SRR11485509-11485628. Raw fastq files were analyzed by Quantitative Insights Into Microbial Ecology (QIIME2-2019.10) (Bolyen et al., 2019) and processed using the Deblur algorithm to denoise and infer exact amplicon sequence variants (ASVs). The curated ASVs were aligned and annotated by the naive Bayes classifier using the SILVA 132-99nb database.

In order to calculate alpha diversity, the complete ASVs count table was rarefied to 71,364 sequences per sample. Microbiota alpha diversity was quantified as community richness by the Shannon index, while microbiome beta diversity was estimated using samples distances by Bray–Curtis. A two-dimensional scatter plot was generated using non-metric multidimensional scaling analysis (NMDS). The bacterial taxonomic distributions of sample communities were visualized. Taxa abundance was estimated by linear discriminant analysis effect size (LEfSe) (Segata et al., 2011).

Predicted microbial metabolic functions were determined by Phylogenetic Investigation of Communities by Reconstruction of Unobserved States (PICRUSt2) as described by Douglas et al. (2020). The 16S rRNA metagenome dataset was used to predict metabolic functions using the Kyoto Encyclopedia of Genes and Genomes (KEGG) and the ortholog classification database at hierarchy level 3 pathways.

### Statistical Analysis

Results are expressed as mean ± SE. The statistical significance of differences between groups was determined with a t-test or ANOVA test, as appropriate, by using GraphPad Prism 7.2 software. Microbiome alpha diversity comparison was done by the Wilcoxon test. Taxa analysis was performed by LEfSe using R package microeco (Segata et al., 2011). Metabolic pathway comparison was determined by ANOVA test using STAMP v2.1.3 (Statistical Analysis of Taxonomic and Function software) (Parks et al., 2014). *p*-Values <0.05 were considered significant.

## Results and Discussion

### Mouse Model Characteristics

#### Infectious Dose

**Figure 1:**
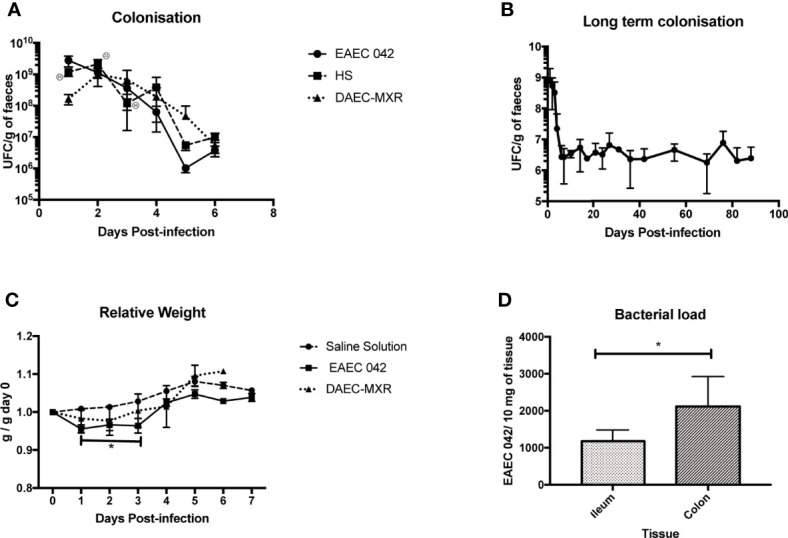
Evaluation and characterization of C57BL/6 mice infected with EAEC 042. **(A)** Colonization of C57BL/6 mice with EAEC 042, DAEC-MXR, and HS at a 5 × 10^9^ dose. Fecal samples were collected and weighed every day in a sterile environment. Bacterial concentration in feces was determined by CFU/g. Colonization results for EAEC 042 are the mean of three independent experiments of five mice each. In the first colonization experiment, five and seven mice were infected with EAEC 042 and HS strains, respectively. In the second experiment, five and six mice were infected with EAEC 042 and DAEC-MXR, respectively. In the third experiment, only EAEC 042 strain was used. No bacterial growth was observed on MacConkey agar plate after culturing feces from control mice from the three experiments, inoculated with saline solution (SS) (N = 12). **(B)** Long-term colonization with EAEC 042; three mice from experiment three were followed up for 90 days. **(C)** Relative weight of mice after inoculation with EAEC 042, DAEC-MXR, and SS. The relative weight was calculated as the day weight post-infection divided by the weight before infection (day 0). Results are the mean of three independent experiments with saline solution (SS) (N = 12) and EAEC 042 (N = 15) and 1 experiment with DAEC-MXR (N = 6), *p < 0.05. **(D)** Intestinal bacteria load. On day 7, post-infection DNA from ileum (N = 5) and colon (N = 7) of EAEC 042-infected mice was obtained. By real-time PCR, *aii*C gene (a specific chromosomic marker of EAEC 042) was screened, *p < 0.05. EAEC, enteroaggregative *Escherichia coli*; CFU, colony-forming unit.

We evaluated the effect of three different infective doses of EAEC 042—5.0 × 10^9^/100 μl, 9.5 × 10^9^/100 μl, and 1.0 × 10^10^/100 μl CFU—on C57BL/6 male mice, aged 6 weeks, which were not infected with commensal *E. coli*, and their microbiota are intact. The optimal dose was 5 × 10^9^/100 μl CFU, since none of the animals died before the first week of infection, as was observed with the other two infectious doses. In contrast, with a weaned male C57BL/6 mouse (21 days old) model, the animals survived to high-challenge EAEC 042 (1.0 × 10^10^/100 μl and 2.0 × 10^10^/100 μl) (Bolick et al., 2013). As illustrated in **Figure 1A**, after inoculation of mice with 5 × 10^9^/100 μl EAEC, it was observed that animals continued shedding for a week at concentrations between 10^5^ and 10^6^ CFU/g of stool (until animals were sacrificed). Our observation is in line with the EAEC 042 weaned mouse model since it was reported that mice continued to shed until day 14 (the experiment’s duration), but the bacterial shedding concentrations were lower (>10^3^ CFU/g) than the ones we observed, even though the initial infectious dose was 0.5 log higher (Bolick et al., 2013). To be sure that an EAEC-infected mouse model was pathogen-specific, C57BL/6 mice were challenged with another human commensal or enterodiarrheagenic *E. coli*. Mice were inoculated with two commensal *E. coli* reference strains (3030 and HS) and an isolate from a Mexican child younger than 1 year with diarrhea, a strain characterized as diffusely adherent *E. coli* (DAEC-MXR), one of the six diarrheagenic pathotypes described. After 24 h post-inoculation with *E. coli* reference strains 3030 or HS with two doses of 1.0 × 10^10^/100 CFU and 5.0 × 10^9^/100 μl CFU, 70% (8/11) and 40% (3/8) of mice died, respectively, whereas none of the mice inoculated with DAEC-MXR (5.0 × 10^9^/100 μl CFU) died.

Furthermore, in order to evaluate if inoculated mice (5 × 10^9^/100 μl) shed for more than 2 months, animal stools were assessed for the presence of EAEC 042 strains every 3 days after the first week and once a week after the first month in all animals shedding of microorganisms persistent in stools until day 90, the experiment’s duration (**Figure 1B**). This observation reveals that EAEC 042 colonizes for long periods in the gut of C57BL/6; correspondingly, children in endemic EAEC regions are frequently colonized with this pathogen without presenting diarrhea (Opintan et al., 2010; Acosta et al., 2016; Havt et al., 2017).

Colonization experiments and morphological *in vivo* and *in situ* changes induced by these two *E. coli* diarrheagenic pathotypes were evaluated to establish pathogen-specific effects.

EAEC 042-infected mice significantly (*p* < 0.05) lost weight during the first 3 days post-challenge in comparison with control mice given saline solution and DAEC-MXR-inoculated mice (**Figure 1C**). Similarly, in an EAEC 042 weaned mouse model, a single oral challenge of EAEC 042 resulted in significant growth shortfalls (5%–8% of body weight in 12 days) (Bolick et al., 2013). DAEC-MXR-inoculated mice remained colonized for a week, which is the experiment duration, and shedding similar concentrations (10^5^ ~10^6^CFU/g stools) to EAEC 042-infected mice.

Bacterial load was evaluated in ileum and colon tissues of five and seven EAEC 042-infected mice, respectively, at day 7 post-challenge, by real-time PCR using the *aaiC* EAEC chromosomal marker. As illustrated in **Figure 1D**, both the ileum and colon were colonized by EAEC 042, 1,177.454 ± SD 300.91 bacteria/10 mg and 1,811.683 ± SD 856.58 bacteria/10 mg of tissue, respectively. The colon gut region colonized by commensal *E. coli* presented a higher bacterial load than the ileum at day 7 post-infection; similar to the EAEC 042 weaned mouse model, bacteria were identified in both regions, and bacterial load was higher in the colon as well (Bolick et al., 2013).

#### *In Situ* and *In Vivo* Characteristics

**Figure 2:**
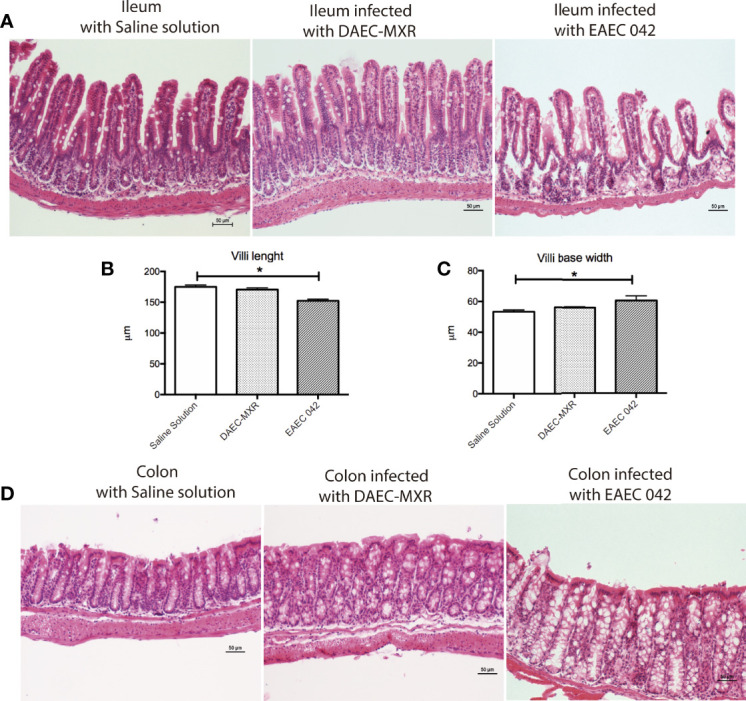
Histopathology of gut infection with EAEC 042 and DAEC-MXR. **(A)** Ileum stained with H&E. Ileum from mice infected with EAEC 042 at day 7 post-infection presented edema zones and shorter and wider villi than control mice and DAEC-MXR. **(B)** Villus length. EAEC 042-infected mouse ileum had significantly shorter villus than saline solution control and DAEC-MXR mice, *p < 0.05. Ileum villus length was measured from tissue section (N = 5 mice per group. **(C)** Villi width. EAEC 042-infected mice had significantly wider villi bases in comparison with control mice and DAEC-MXR-infected mice. **(D)** Colon stained with H&E. An increased mucus secretion and cell shedding were observed in the colon of EAEC 042-infected mice in comparison with control mice. EAEC, enteroaggregative *Escherichia coli*.

**Figure 3:**
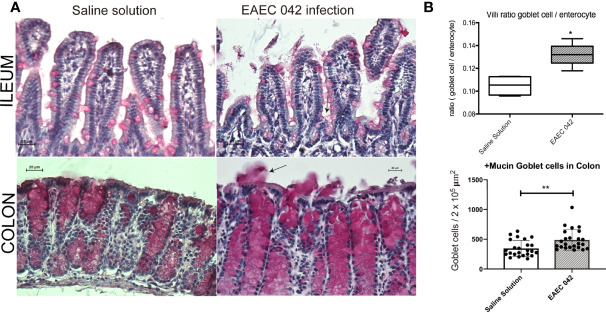
Mucus secretion by mice infected with EAEC 042. **(A)** Ileum and colon stained with periodic acid-Schiff (PAS). Mucus mucopolysaccharide was stained in pink inside and outside the goblet cells. Increased secretion of mucus was observed in colon of infected mice with EAEC 042. Arrow indicates the mucus secreted to the lumen by the goblet cells. **(B)** Ileum villus and crypt ratios between goblet cell/enterocyte and colon goblet cell/area. Goblet and enterocyte cells were counted along the ileum (villi and crypts). Colon goblet cells were counted from 2 × 10^5^ µm^2^ of tissue area. The values represent the mean ± SD, N = 4 saline solution (control mice), 5 EAEC 042-infected mice, *p < 0.05, **p ≤ 0.01. EAEC, enteroaggregative *Escherichia coli*.

Ileum and colon morphological changes were evaluated by H&E-stained tissues on control mice and EAEC 042- and DAEC-MXR-infected mice after 7 days post-infection (**Figure 2**). EAEC 042-infected mice revealed more notable alterations in the ileum than in colon tissues (**Figure 2**), ileal villi were shorter with wider bases and edema formation, and cell desquamation was observed, in comparison with ileum tissues of both control mice and DAEC-MXR-infected mice. PAS-stained ileum and colon tissues of EAEC 042-infected mice, sacrificed at 7 days post-infection, revealed increased mucus production in both tissues than in control mice (**Figure 3**). Furthermore, on infected ileum and colon tissues, a significantly higher ratio of goblet cells/enterocytes (*p* < 0.05) and number of positive mucins goblet cells were observed, respectively, as compared with control mice. It is possible that the observed increase in mucus production is due to the presence of Pic, which has been shown to induce mucus production, accompanied by an increase in the number of mucus-containing goblet cells in an *in vivo* ileal rat loop system (Navarro-Garcia et al., 2010). Of interest, in this system, the isogenic 042 *pic* mutant was unable to cause mucus hypersecretion (Navarro-Garcia et al., 2010). These findings are in line with those reported in patients infected with EAEC 042 who usually have watery mucus diarrheal episodes (Estrada-Garcia and Navarro-Garcia, 2012).

Overall, the most pronounced morphological changes were observed in the ileum, suggesting that pathogenic effects of EAEC 042 are more pronounced in this tissue than in the colon; notably, the most important physiological function of the ileum is nutrient absorption, perhaps suggesting a mechanism of growth shortfalls seen in tEAEC-infected patients. In conjunction, the ileal morphological changes and mucus production in EAEC 042 infection may compromise host nutrient absorption. It has been observed that tEAEC infection among children is associated, even in the absence of diarrhea, with growth shortfalls (Opintan et al., 2010) and with significantly greater height-for-age Z-score (HA-Z) decline (*p* < 0.05), supporting the pathogenic role of tEAEC strains in ileum dysfunction in human.

#### Enteroaggregative *Escherichia coli* 042-Infected Mouse Gut Microbiota Characterization

**Figure 4:**
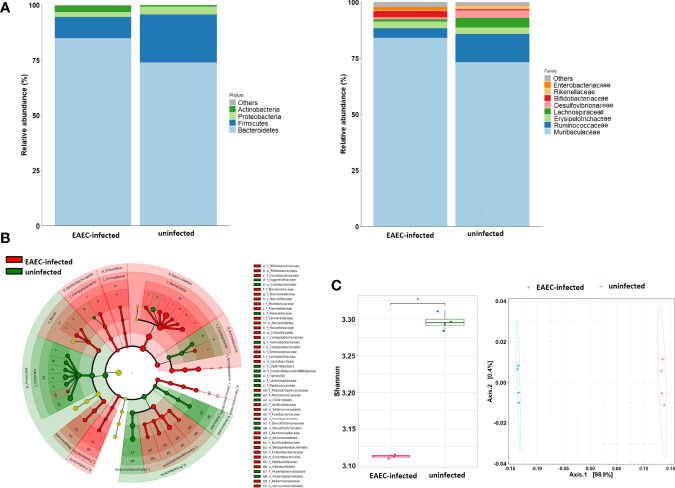
Impact of EAEC 042 infection on the gut microbial community at 21 days post-challenge. **(A)** Community profile at phylum and family taxonomic levels. Taxa units were organized based on the most abundant taxa; fewer representative taxa were grouped as others. **(B)** Classification tree of mice EAEC 042-infected (red color) and uninfected (green color) bacterial communities based on LEfSe analysis, with sizes of nodes reflecting relative abundances. **(C)** Alpha diversity metrics were compared between EAEC 042-infected and uninfected mice using Shannon indices, and beta diversity analysis was performed using NMDS. Each group (N = 4), *p < 0.01, versus uninfected mice. EAEC, enteroaggregative *Escherichia coli*; LEfSe, linear discriminant analysis effect size; NMDS, non-metric multidimensional scaling analysis.

A total of 862,638 high-quality paired sequences were obtained from stools of uninfected and EAEC 042-infected mouse samples, with an average sequencing depth of 107,829 (range 71,364–144,484), which were clustered into 825 ASVs and classified into 36 bacterial groups at the family level. The gut bacteriota phylum and family compositions of EAEC 042-infected and uninfected mice are shown in **Figure 4A**. The hierarchical tree constructed based on the relative abundance of taxonomic levels (phylum, class, and family) by LEfSe (LDA > 2.5) revealed that the phylum Bacteroidetes and its families Lactobacillaceae, Muribaculaceae, Prevotellaceae, Marinifilaceae, and Tannerellaceae, plus the class Gammaproteobacteria and its families Aeromonadaceae, Burkholderiaceae, Enterobacteriaceae, and Pasteurellaceae, were significantly increased among EAEC 042-infected mice in comparison with uninfected mice (**Figure 4B**). As illustrated in **Figure 4C**, the Shannon index alpha diversity (between samples of a group) significantly decreased among EAEC 042-infected mice, and the NMDS beta diversity (EAEC 042-infected vs. uninfected mice) was significantly different between groups. Functional analysis of the gut bacteriota of EAEC 042-infected mice by 2 KEGG revealed enrichment of two pathways described as “metabolism of cofactors and vitamins” and “biosynthesis and metabolism glycan” (**Supplementary Figure 1**). Further characterization of the later pathways by 3 KEGG indicated that in particular, it enriched the folate biosynthesis, biotin metabolism, lipopolysaccharide biosynthesis, vitamin B6 metabolism, and other glycan degradation pathways. Altogether, our results clearly indicate that EAEC 042-infected mice have a dysbiotic bacteriota. Of interest, class Gammaproteobacteria increases in the bacteriota of antibiotic-treated mice (Sekirov et al., 2008; Barreto et al., 2020), and animals are more prone to *Salmonella enterica* serovar Typhimurium infection (Sekirov et al., 2008). Similar to our observations, mice infected with the tEAEC-STEC O104:H4 C227-11 strain had an imbalance of bacteriota, manifested as an increase in γ-Proteobacteria and *Lactobacillus* spp., with direct alteration in intestinal metabolites driven by microbiota changes (Machado Ribeiro et al., 2021).

### Expression and Localization of Intestinal Host Molecules ZO-1 and β-Catenin in Enteroaggregative *Escherichia coli* 042-Infected Mice

**Figure 5:**
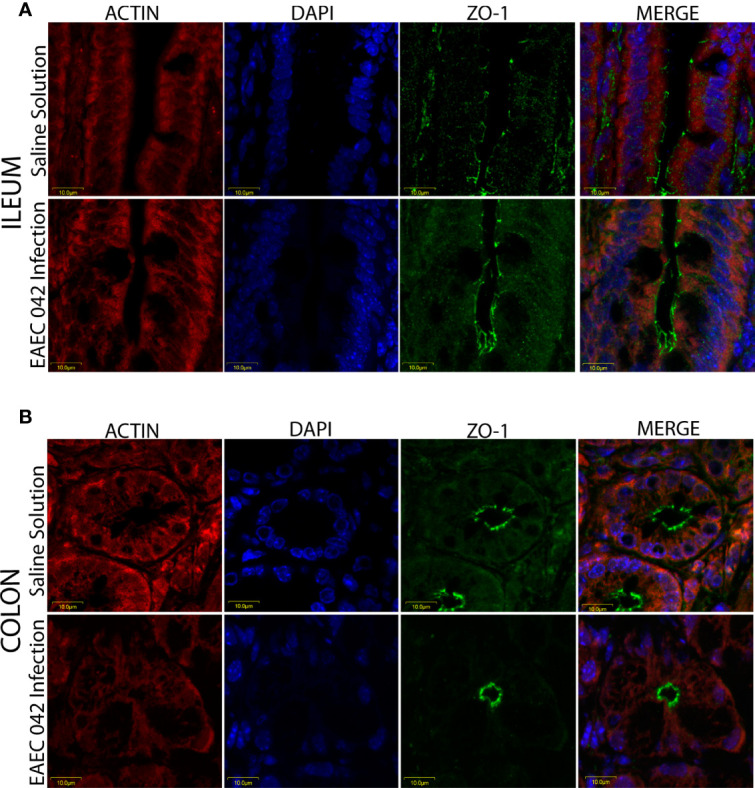
Immunofluorescence of the tight junction protein ZO-1 at 7 days post-infection. **(A)** Ileum of mice with EAEC 042 infection and control mice with Saline Solution. **(B)** Colon of mice with EAEC 042 infection and control mice with Saline Solution. ZO-1 was stained with a secondary antibody conjugated with Alexa 480 antibody (green), actin with an F-actin probe Alexa 568 phalloidin (red), and nucleic acids with DAPI (blue).

**Figure 6:**
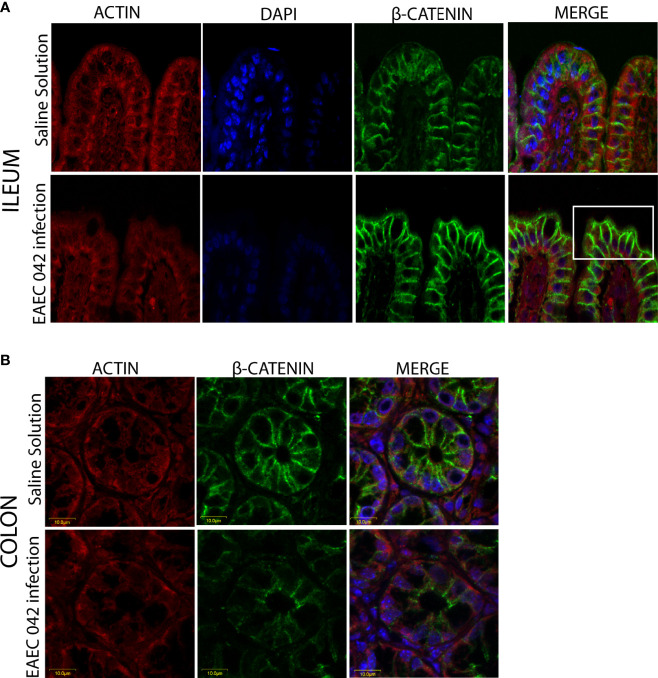
Immunofluorescence of the adherent junction protein β-catenin at 7 days post-infection. **(A)** Ileum of mice with EAEC 042 infection and control mice with Saline Solution. **(B)** Colon of mice with EAEC 042 infection and control mice with Saline Solution. β-Catenin was stained with a secondary antibody conjugated with Alexa 480 antibody (green), actin with an F-actin probe Alexa 568 phalloidin (red), and nucleic acids with DAPI (blue).

The effect of EAEC 042-infection on the gut barrier was investigated by evaluating the expression of ZO-1 tight junctions and β-catenin adherence junction proteins, on the ileum and colon, after 7 days post-challenge and uninfected mice (control group) by immunofluorescence. As illustrated in **Figure 5**, ZO-1 localization remained unaltered in both ileum and colon tissues, suggesting that tight junctions are not affected at this stage of EAEC 042 infection and confirming that tEAEC, which is not an invasive pathogen, does not disrupt the epithelial barrier integrity as do *S. enterica* serovar Typhimurium and *Shigella flexneri*, both invasive bacteria (Tafazoli et al., 2003; Meza-segura et al., 2021). On the other hand, EAEC 042 infection influenced β-catenin expression in the ileum since it was observed not only in the latero-basal cell region, adherence junction, and control tissues but also in the apical cell region (**Figure 6**). In contrast, in colon-infected tissues, β-catenin expression in the latero-basal cell region was diminished in comparison with control colon tissues; however, discrete β-catenin expression was observed in the apical cell region as well (**Figure 6**). Overall, our results reveal that at 7 days post-EAEC 042 infection, β-catenin expression is differential in ileum and colon tissues. So far, it is not clear if EAEC 042 infection and pathogenesis in humans take place in the ileum or colon, but a significant effect on β-catenin expression observed on ileum tissues suggests that at least in the EAEC 042 murine model, infection impacts ileal tissues more than the colon.

### Role of the AAF-II Fimbria in Enteroaggregative *Escherichia coli* 042 Infection

**Figure 7:**
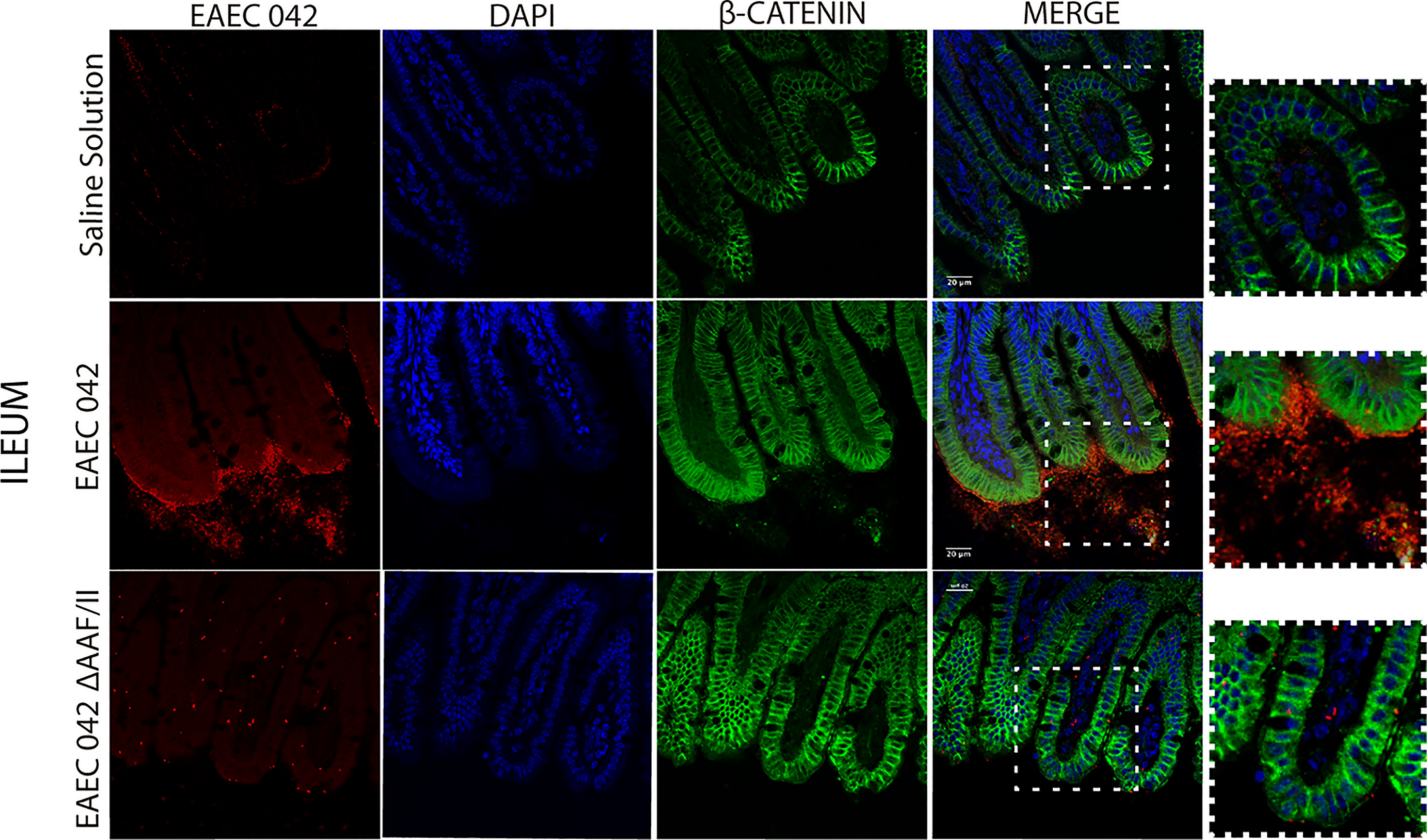
Immunofluorescence of β-catenin in ileum at 4 days post-infection. β-Catenin was stained with a secondary antibody conjugated with Alexa 480 antibody (green), bacteria with a primary antibody against EAEC 042 AAF/II and as secondary antibody Alexa 568 (red), and nucleic acids with DAPI (blue).

**Figure 8:**
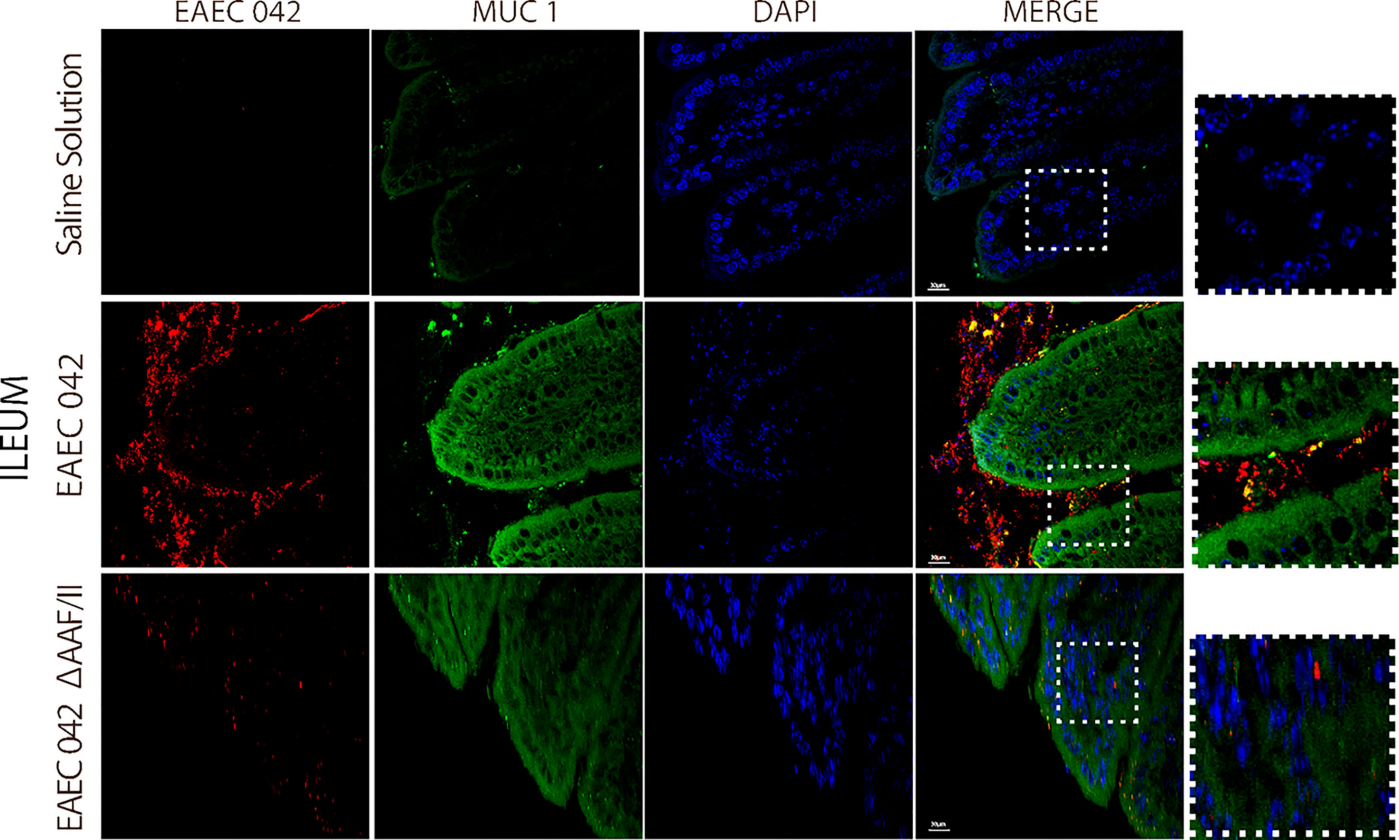
Immunofluorescence of MUC1 on ileum at 4 days post-infection. MUC1 was stained with a secondary antibody conjugated with Alexa 480 antibody (green), bacteria with a primary antibody against EAEC 042 AAF/II and as secondary antibody Alexa 568 (red), and nucleic acids with DAPI (blue).

Since EAEC 042 AAF/II plays an important role in pathogenesis, its role during infection was evaluated using EAEC 042 wild-type and EAEC 042 ΔAAF/II mutant strains and antibodies increased against the mutant strain that also recognizes 042 wild-type bacteria. Since growth shortfalls were observed among infected mice at the beginning of the infection (**Figure 1**), ileal tissues were evaluated for the bacterial presence and β-catenin expression at this stage of infection. As illustrated in **Figure 7**, at 4 days post-infection, the wild-type EAEC 042 strain seems to form a biofilm on the apical surface of the ileal villi, as is shown with the red fluorescent dye. In contrast, no biofilm formation was observed on the ileum of EAEC 042 ΔAAF/II-infected mice, and only very few bacteria were observed on the ileum surface (**Figure 7**). Biofilm formation by strain 042 has been demonstrated in the neonatal mouse model as well (Roche et al., 2010). At 4 days post-challenge, the β-catenin expression on the ileum of EAEC 042 mice was latero-basal and apical, like the expression observed at 7 days post-infection (**Figure 6**), in contrast with the animals infected with the EAEC 042 ΔAAF/II where β-catenin expression was similar to that observed on the ileum of control mice (**Figure 7**). In 2017, Boll et al. proposed that MUC1, epithelial transmembrane mucin, is the cell receptor for EAEC 042 strain and that AAF/II mediates this interaction on T84 human colonocytes, facilitating bacterial adhesion (Boll et al., 2017). Therefore, using the newly developed mouse model, the expression and distribution of MUC1 on ileum tissues of EAEC 042, EAEC 042 ΔAAF/II-infected mice, and control mice at 3 days post-infection were evaluated. As illustrated in **Figure 8**, once more, a biofilm formation was observed on the apical surface of the ileal villi of mice infected with EAEC 042 and none on the ileum of EAEC 042 ΔAAF/II-infected mice, as previously observed on day 4. It seems that in comparison with control and EAEC 042 ΔAAF/II mice, MUC1 is highly expressed on the surface of the ileal tissues of EAEC 042-infected mice. Notably, on the ileal tissues of EAEC 042-infected mice, we observed colocalization of MUC1 and EAEC 042 bacteria (yellow dots), but not on the ileum of mice infected with the AAF/II mutant.

Together, our results suggest that EAEC 042 strain forms a biofilm on the apical surface of ileal villi of infected mice, and it seems to be dependent on the AAF/II. So far, *in vitro* experiments have demonstrated the importance of EAEC 042 AAF/II in bacteria adherence assays to T84 cells (Gonyar et al., 2020). Furthermore, to the best of our knowledge, it is reported for the first time that the 042 EAEC strain induces the expression of β-catenin on the apical surface of ileum enterocytes *in vivo*, which may be a result of the biofilm and/or a result of AAF/II fimbria interaction with a surface molecule. Due to the colocalization between EAEC 042 strain and MUC1, it seems that MUC1 may as well be the AAF/II receptor in mice, as previously shown in *in vitro* experiments with human T84 cells (Boll et al., 2017). Furthermore, EAEC 042 infection enhances MUC1 expression in human fetal small intestinal xenografts in an AAF/II-dependent manner (Boll et al., 2017).

On the other hand, the expression of β-catenin on the apical surface of ileum enterocytes, only among animals infected with the EAEC 042 wild type and not in animals infected with EAEC 042 AAF/II-mutant, suggests that potentially AAF/II fimbria directly or indirectly participates in β-catenin expression and localization *in vivo*. It has been reported that the cytoplasmic tail of MUC1 competes with E-cadherin for binding to β-catenin in human colon carcinoma HCT116/V cells (Ren et al., 2002). Furthermore, it has been reported that in human AGS gastric adenocarcinoma cells infected with *Helicobacter pylori*, MUC1 formed a co-immune precipitation (co-IP) complex with β-catenin and the CagA virulence factor, where MUC1 overexpression reduced CagA/β-catenin co-IP (Guang et al., 2012). In addition, in the absence of MUC1 overexpression in AGS cells, *H. pylori* infection increased the nuclear level of β-catenin, whereas MUC1 overexpression decreased bacteria-driven β-catenin nuclear localization (Guang et al., 2012). Therefore, it is plausible that in EAEC 042 infection in mice, AAF/II fimbria binds to MUC1 present on the enterocyte surface, inducing β-catenin expression, which binds to the cytoplasmic tail of MUC1 enterocytes at the apical surface. On the other hand, it has been demonstrated that β-catenin is a transcriptional factor that has been proven to induce the expression of IL-8 genes in *H. pylori*-infected AGS cells (Guang et al., 2012; Ma and Hottiger, 2016). It has been reported that EAEC 042 induces *in vitro* production of IL-8, and its polymorphisms are associated with different clinical outcomes among EAEC-infected patients and subjects with EAEC-associated diarrhea; individuals with AA IL-8 genotype at position 251 produce greater concentrations of fecal IL-8 than those with the AT or TT genotypes (Steiners et al., 1998; Jiang et al., 2003).

## Conclusion

A novel mouse model of the EAEC 042 strain has been developed, which mimics human infection, but not a diarrheal disease. EAEC 042 mice infection induced intestinal bacterial dysbiosis and mucus secretion, plus biofilm formation on the ileal surface. Furthermore, the model revealed the important role of the AAF/II fimbria in infection and pathogenesis in ileal enterocytes; AAF/II may mediate binding of host receptors as MUC1 and may induce expression and localization of the β-catenin adherence junction host protein and transcriptional activator factors. The mouse model provides an excellent tool to characterize the mechanisms by which EAEC exerts its pathogenic effects *in vivo* and *in situ*.

## Data Availability Statement

The datasets presented in this study can be found in online repositories. The names of the repository/repositories and accession number(s) can be found below: https://www.ncbi.nlm.nih.gov/, PRJNA809099.

## Ethics Statement

The animal study was reviewed and approved by CINVESTAV Ethics Committee, # 254994.

## Author Contributions

NM-G and TE-G conceived the experiments. NM-G, MM-S, CL-S, AB, SG-G, and JG-B performed the experiments. AB and AM-T performed all bioinformatics analyses. NM-G and FH-C performed the statistical analyses. FH-C worked on the quality of the images and figures. VT, MS, and JN provided guidance, resources, and input. NM-G and TE-G wrote the manuscript, which was reviewed and approved by all authors. All authors contributed to the article and approved the submitted version.

## Funding

CONACYT scholarship 368716 was awarded to NM-G, 368026 to MM-S, 483617 to AB, and 780260 to FH-C. TE-G was a recipient of CONACYT Grant 254994. MS received funding from Consejo Nacional de Ciencia y Tecnología (CONACYT; grant CB-2016-284292).

## Conflict of Interest

The authors declare that the research was conducted in the absence of any commercial or financial relationships that could be construed as a potential conflict of interest.

## Publisher’s Note

All claims expressed in this article are solely those of the authors and do not necessarily represent those of their affiliated organizations, or those of the publisher, the editors and the reviewers. Any product that may be evaluated in this article, or claim that may be made by its manufacturer, is not guaranteed or endorsed by the publisher.

## Supplementary Material

Supplementary Figure 1 Prediction of functional microbial metabolic pathways using PICRUSt analysis. The abundance of statistically significant metabolic pathways **(A)** KEGG level 2 and **(B)** KEGG level 3 between mice EAEC-infected (red colour) and uninfected (green colour) bacterial communities. All statistically significant pathways (p < 0.05).
